# Graves' disease with pancytopenia: A rare case report

**DOI:** 10.1002/ccr3.8312

**Published:** 2024-01-05

**Authors:** Neda Meftah, Sadegh Sedaghat, Soghra Rabizadeh, Sara Seifouri, Khatereh Nasirimeh, Manouchehr Nakhjavani

**Affiliations:** ^1^ Department of Internal Medicine, School of Medicine Babol University of Medical Sciences Babol Iran; ^2^ Endocrinology and Metabolism Research Center (EMRC) Vali‐Asr Hospital Tehran University of Medical Sciences Tehran Iran

**Keywords:** case report, Graves' disease, pancytopenia, thyrotoxicosis

## Abstract

**Key Clinical Message:**

In this case report we describe a patient with Grave's disease (GD) who was first diagnosed with pancytopenia and did not have any typical symptoms of GD. His hematologic abnormalities were alleviated after treatment with an anti‐thyroid drug. Hence, in patients with pancytopenia, GD should also be considered.

**Abstract:**

A variety of hematologic abnormalities can be seen in Graves' disease (GD), however; here, we describe a patient with GD and a very rare complication; pancytopenia. His hematologic abnormalities and clinical status were alleviated after treatment with an anti‐thyroid drug. Hence, in patients with pancytopenia and normal bone marrow examination, GD should also be considered.

## Graphical abstract

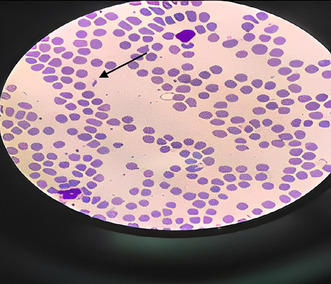

## 1 INTRODUCTION

Graves' disease (GD is a chronic autoimmune disorder that produces antibodies against the thyrotropin (TSH) receptors on the thyroid.^1^ GD is a syndrome that is characterized by hyperthyroidism with a diffuse goiter, an eye disease that involves intra‐orbital structures, and dermopathy known as pretibial myxedema.^2^ GD can cause a variety of symptoms such as hyperactivity, hyper defecation, weight loss with increased appetite, weakness, and polyuria.^3^ In about half of GD patients, the characteristic clinical hallmarks may be absent therefore other findings may help us in the diagnosis of GD.^4^ Sometimes, GD may present with single‐cell lineage hematological abnormalities such as neutropenia, anemia, and thrombocytopenia.^3^, ^5^ But Pancytopenia due to GD is a rare complication.^5^, ^6^ Although the pathogenesis of this association is not clearly fathomed, hypotheses based on immunogenic and toxic mechanisms have been considered.^7^ The lack of production of hematopoietic cells in the bone marrow and the increase in the immunologic destruction of mature hematopoietic cells can be considered the two main pathways leading to pancytopenia.^1^

## 2 CASE PRESENTATION

A 41‐year‐old man presented with fatigue and a weight loss of approximately 10 kg within 2 months. He did not take any medications except acetaminophen for his occasional headaches and had no significant medical history. He was not a smoker but had a history of alcohol consumption about twice a week. His father died due to lung cancer. There was no family history of thyroid or autoimmune disease.

On physical examination, his weight was 65 kg and his height was 182 cm. His vital signs included the following: blood pressure of 105/60 mmHg, temperature of 37.1°C, pulse rate of 90 beats/min, and respiratory rate of 15 beats/min.

He had a depressed appearance. There was no sign of Hair or skin changes. There was no lymphadenopathy or hepatosplenomegaly, and his cardiovascular and respiratory systems examinations were unremarkable.

**FIGURE 1:**
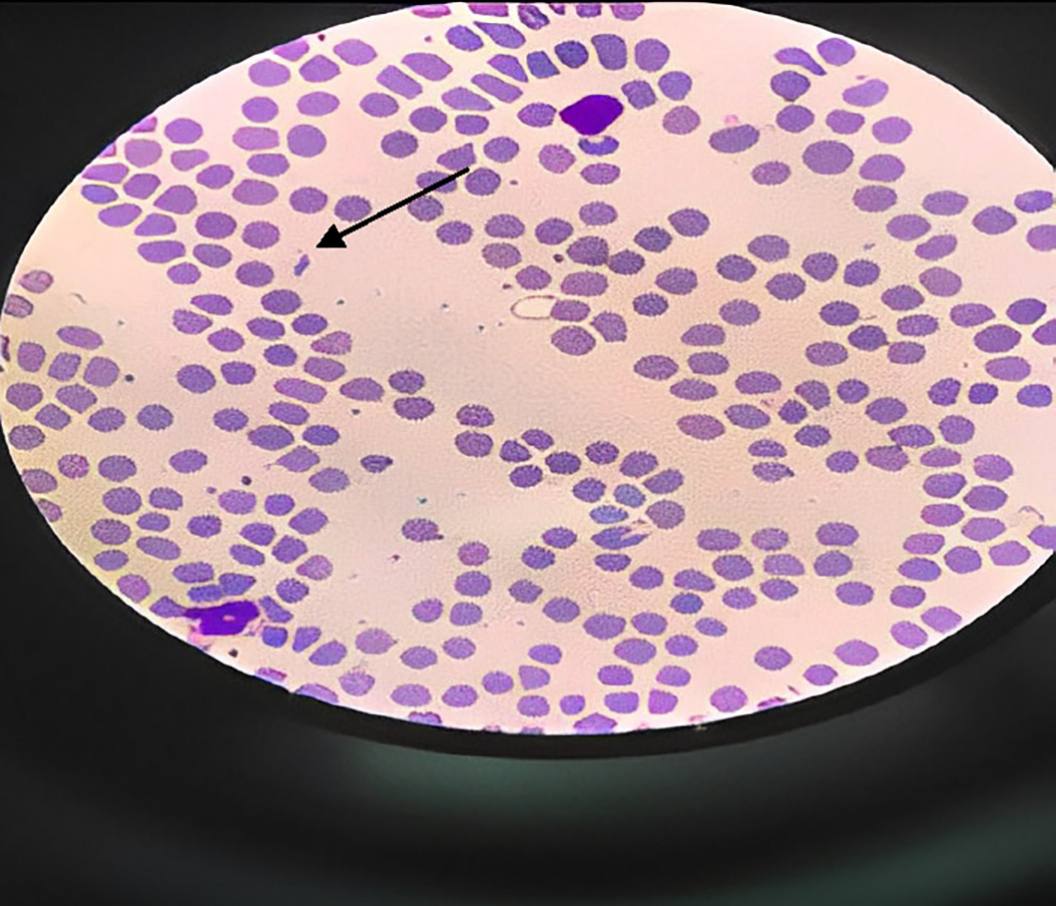
Peripheral blood smear shows mild anisocytosis with large platelet. Large platelet is shown with an arrow. (Wright‐Giemsa stain, Magnification: 100×).

Initial biochemical analysis showed leukopenia (white blood cell count 2.4 × 103/μL, neutrophil count 36%, lymphocyte count 63%, and monocyte count 1%), normocytic normochromic anemia (hemoglobin 11 g/dL, Red blood cell count 5.1 million cells per microliter, hematocrit 32.8%, mean cell volume 82.2 fL, and mean cell hemoglobin concentration 27 g/dL), and thrombocytopenia (platelet count 70 × 103/μL) (Figure 1A). There was mild anisocytosis in the peripheral blood smear (Figure 1). The reticulocyte count was 2%, serum iron was 75 μg/dL, total iron binding capacity was 320 μg/dL, serum ferritin was 100 ng/mL, serum vitamin B12 was 358 ng/mL (normal, 200–900 ng/mL)), and serum folate was 5.8 ng/mL (normal, >2 ng/mL). Laboratory test results are shown in Table 1. His laboratory findings did not manifest any evidence of infection or abnormalities in electrolytes, liver, and renal function tests.

**TABLE 1:** Laboratory case results.

Laboratory tests	Conventional units	Reference range values	Patient results (2020)
White blood cell count	×103/μL	4.5–10.5	2.4
Neutrophil count	%	40–60	36
Lymphocyte count	%	20–40	63
Monocyte count	%	2–8	1
Red blood cell count	Million cells Per microliter (μL)	4.35–5.65	5.1
Hemoglobin	g/dL	13.8–17.2	11
Hematocrit	%	40–54	32.8
MCV	fL	80–100	82.2
MCHC	g/dL	27–33	27
Platelet count	×103/μL	150–450	70
Reticulocyte count	%	0.5–2.5	2
Serum Iron	μg/dL	65–176	75
Total Iron binding capacity	μg/dL	255–450	320
Serum ferritin	ng/mL	24–336	100
Serum vitamin B12	ng/mL	200–900	358
Serum folate	ng/mL	>2	5.8
Bone marrow cellularity	%	30–70	50
TSH	μIU/mL	0.5–5	0.004
T4	μg/dL	4.7–12.5	24
T3	ng/mL	0.8–2.3	6

Abbreviations: MCHC, mean cell hemoglobin concentration; MCV, mean cell volume; T4, thyroxine; T3, triiodothyronine; TSH, thyroid stimulating hormone.

**FIGURE 2:**
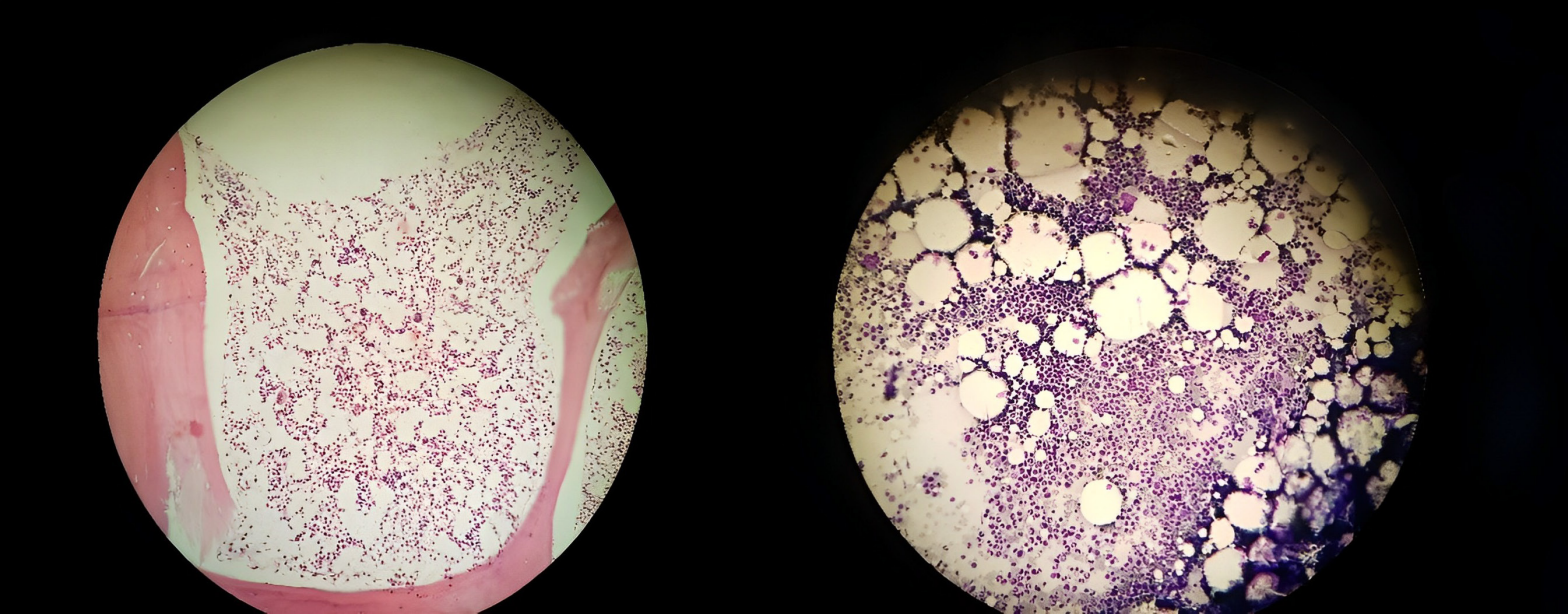
(A) Bone marrow aspiration smear shows normocellular marrow and normal hematopoiesis (Wright‐Giemsa stain). (B) Bone marrow biopsy shows about 50%cellularity (H&E stain).

Bone marrow aspiration smears indicated a normocellular marrow. Megakaryocytes and their precursors were adequate in number. The erythroid and myeloid series were completely matured (Figure 2). Bone marrow biopsy showed a cellularity of about 50%. Therefore, pancytopenia was initially assumed to be caused by an immunologic mechanism or a bone marrow stem cell disorder such as immune‐based. Bone marrow stem cell disorder like myelodysplastic syndrome was excluded because bone marrow was normal. While hematological aspects were being debated, the results of thyroid function tests came showing a significantly declined TSH (0.004 μIU/mL) with a raised T4 24 μg/dL (normal: 4.7–12.5) and T3 6 ng/mL (normal: 0.8–2.3) indicating the presence of marked hyperthyroidism.

Retrospective clinical examination manifested mild diffuse goiter as well as fine tremors of the outstretched fingers. Unfortunately, these findings had been missed in the earlier clinical examinations. Later, Graves' disease was diagnosed via investigating with thyroid scanning with technetium 99 m pertechnetate, showing a diffuse rise in uptake. Treatment with methimazole (30 mg/day) was commenced. After 2 days of treatment, the platelet count and white blood cell count began to slowly rise. After 6 days, he was discharged from the hospital with acceptable improvement in his condition. He continued to take 30 mg/day of methimazole and was followed at the outpatient clinic of the hospital. After 2 weeks of treatment, TT4 and TT3 levels decreased gradually. The same dose of methimazole was continued and after 4 weeks, the clinical condition became better. Blood counts and FT4 reached normal levels after 4 and 8 weeks of treatment, respectively. Because of the risk of ATD‐induced pancytopenia, he was further treated with radioiodine ablation (Iodine‐ 131).

## 3 DISCUSSION

In this case, the patient presented with pancytopenia and hyperthyroidism. He had no history of drug consumption or infection‐related laboratory findings. A bone marrow smear ruled out the possibility of an underlying myelodysplastic syndrome. These findings suggest that hyperthyroidism can induce pancytopenia. After the improvement in the thyroid hormone levels, the blood counts were normalized promptly. Single‐lineage hematologic abnormalities are more common in patients with GD but pancytopenia has been rarely described in the literature.^1^, ^8^, ^9^ Anemia has been found in 33% of GD and a patient's red blood cell mass can also increase in GD, due to both thyroid hormones' direct effects on the erythroid marrow and a rise in the production of erythropoietin. However, anemia can also be detected in hyperthyroid patients generally due to concomitant plasma volume expansion, the decline in erythrocyte lifespan, abnormal iron use, lack of iron storage, and rising need for vitamin B12 and folic acid. Any type of anemia might encounter in the course of hyperthyroidism. Normocytic anemia is the most common, while microcytic or macrocytic anemia can also occur.^10^

Our patient had normocytic normochromic anemia in the peripheral blood smear. He had no evidence of vitamin B12, iron deficiency, or hemolytic anemia. In bone marrow aspiration and biopsy, bone marrow cellularity and maturity were both normal, and aplastic anemia and myelodysplastic syndrome were both ruled out.

In GD, leukopenia is commonly found as a result of decreased neutrophil counts. Leukopenia can happen as a result of one of these two pathways. First granulopoiesis is reduced by a decrease in the granulocyte reserve of bone marrow, on the other hand, there is a hypothesis that destruction can happen as a result of immunologic mechanism, as antineutrophil antibodies in the serum of patients with thyrotoxicosis were detected.^5^ It is showed that there is a significant association between T3 levels and absolute neutrophil counts, TSH and absolute CD4 counts, T4 levels and absolute CD4 counts.^4^ A characteristic blood finding of GD, called Kocher's blood picture, consists of a rise in lymphocyte counts alongside a normal or marginally low white blood cell count.^11^, ^12^ It has been said that low neutrophil count in thyrotoxicosis is due to a decline in the number of granulocytes in bone marrow as well as reduced circulation duration of these cells. Additionally, cross antigenicity among human TSH receptors and polynuclear neutrophils has been considered a potential cause of neutropenia in thyrotoxicosis.^12^ In the presented patient, Blood findings were compatible with Kocher's blood picture.

Platelet counts are usually normal in GD. Mild immune thrombocytopenia is known to be associated with Graves' disease and alleviated with proper management. Elevating thyroid hormones result in increased destruction of the platelets as well as activation of the reticuloendothelial system resulting in a significant decline in platelets' lifespan. Also, anti‐platelet antibodies were detected in Graves ‘disease. Commonly platelet counts return to normal with proper management.^13^

Pancytopenia can happen as a result of other reasons of thyrotoxicosis and not only Grave's disease. In a study, it showed that one of the underlying pathologies is that high amount of thyroid hormone in blood and its toxic effect on bone marrow cells can lead to increase in functional activity of reticuloendothelial cells, causing insufficient erythropoiesis. The sequestration of immature erythroid cells can lead to all types of anemia by dramatically increasing the intake of vitamin B12, iron, and folic acid (Table 2).^17^

**TABLE 2:** Case reports of Grave's disease presenting as pancytopenia.

Authors	Year	Age	Sex	Origin	Clinical features	Lab results	Hematological findings in peripheral blood and bone marrow	Treatment
Scappaticcio et al.^14^	2020	51	F	Caucasian	Lower abdominal pain, nausea, intermittent vomiting, profound weight loss, Fatigue	TSH <0.01 μIU/mL, FT3 20 pg/mL, FT470pg/mg, TRAB 8 IU/L, (Serum iron, haptoglobin, ferritin, folic acid, and vitamin B12 were normal. Electrophoresis of hemoglobin was normal, direct and indirect coombs tests were negative, slightly hepatosplenomegaly	A peripheral blood smear confirmed the pancytopenia with Lymphomonocytosis (lymphocytes 49% and monocytes 12%), leucopenia, neutropenia. Bone marrow aspiration was refused by patient	Low dose methymasole
Lima et al.^15^	2006	71	M	Brazil	Weakness, Cutaneousmucous pallor	Hemoglobin: 7.3 g/dL, Hematocrit:21.8%, MCV: 82.1 fL, MCHC:27.3 g/dL Direct and indirect coombs tests were negative, increased free thyroxine(FT4: 2.87 ng/dL, decreased thyrotropin(TSH: 0.07 μIU/mL), TgAb: 1276 IU/mL, TPOAb: 436 IU/mL	Slight hypercellularity of the erythroid, granulocytic, and megakaryoblastic lineages, with slight cellular atypia and arrested hematopoiesis, were detected by cytologic and histologic bone marrow evaluations, leukopenia, neutropenia and thrombocytopenia in peripheral blood.	MMI
Lima et al.^15^	2006	35	F	Brazil	Goiter and weight loss, cutaneous‐mucous pallor, a diffuse goiter and jaundice	The hematologic examination showed microcytic and hypochromic anema (Hb: 9.5 g/dL, Hct: 30.2%), FT4 (5.9 ng/dL), TSH (0.01 μIU/mL), TgAb (321 IU/mL), TPOAb (1276.0 IU/mL). ALT, AST <total bilirubin were increased	The cytologic and histologic evaluations of bone marrow revealed slight hypercellularity Leukopenia, neutropenia and thrombocytopenia in peripheral blood.	6 months of MMI
Lima et al.^15^	2006	39	M	Brazil	Tremor in the hands, weight loss, tachycardia, diffuse goiter, exophthalmos	Normochromic and normocytic anemia (Hb:11.9 g/dL, Hct:35.8%).FT4 (7.77 ng/dL), TSH (0.01 μIU/mL) TgAB (121 IU/mL) TPOAb (1000 IU/mL).Direct and indirect tests were negative	Leukopenia, neutropenia and thrombocytopenia were found on peripheral blood.	MMI
Lima et al.^15^	2006	18	F	Brazil	Mucous‐cutaneous pallor, tachycardia, exophthalamus, diffuse goiter, tremor in the hands	FT4 (2.48 ng/dL), TSH (<0.001 μIU/mL), TPOAb (84.6 IU/mL), TgAb (negative), direct and indirect tests were negative. Normochromic and normocytic anemia (Hb (4 g/dL), Hct: (12.4%))	Slight hypercellularity of the erythroid, granulocytic and megakaryoblastic lineages in histologic analysis of bone marrow. Leukopenia, thrombocytopenia and neutropenia in prepheral blood	After therapy with methimazole, the patient evolved to aplastic anemia. then patient underwent radioiodine therapy, standard immunosuppressive treatment with anti‐thymocyte globulin
Garla Et al.^1^	2018	27	M	USA	Sweating, palpitations, heat intolerance, insomnia, and weight loss. Tachycardic, tremors of upper extremities	Suppressed thyroid‐stimulating hormone, high free thyroxine and positive thyroid receptor antibodies. Direct and indirect Coombs tests were negative. Hemoglobin: 9.5 g/dL	Anemia, leucopenia and thrombocytopenia in peripheral blood	Methimazole, propranolol and hydrocortisone after discharge patient had still improvement in his symptoms, therefore thyroidectomy was done.
Bao Fu Et al.^4^	2020	35	F	China	Tenderness on abdominal palpation, goiter, pretibial myxedema without orbitopathy	Hemoglobin was 7.9 g/dL, thyroid function test showed TSH was 0.011 μIU/mL, FT3 was 30.8 pmol/L, FT4 was 116.6 pmo/L, TPOAb was above 600 IU/mL, thyroglobulin antibody was 2567 ng/mL (reference range: 3.5–77) and TRAb was 25.78 IU/L	The blood test result showed white blood cell levels was 2.75 × 10^1^/L(normal range 4–10 × 10^1^/L), the absolute value of neutrophils was lower than normal range: 0.85 × 10^1^/L, platelet count was lower than normal range 16 × 10^1^/L Bone marrow biopsy smear showed megakaryoplasia of bone marrow, the number of thrombocytogenic megakaryocyte was less.	The patient received endotracheal intubation, ventilator, antithyroid drugs, and hormone therapy
Hegazi Et al.^16^	2008	43	F	Kuwait	Dizzinees, dyspnea, bilateral lower limb edema7	Low Hemoglobin (73 × 10^1^/L), low TSH (0.01 μ IU/mL), highT4 (64.08 pmol/L) Serum vitamin B12 and folate was normal	Trephine biopsy was done for bone marrow examination. The results showed hypercellular marrow with normoblastic hyperplasia, normal granulopoiesis and normal megakaryopoiesis. A blood smear showed a microcytic hypochromic picture. Low white blood cell(3.2 × 10^1^/L) low platelet(55 × 10^1^/L) Microcytic hypochromic anemia	Carbimazol (30 mg/day), blood transfusion for anemia and antibiotic cover for neutropenia

There are some reports regarding hyperthyroid patients who present with pancytopenia.^1^, ^4^, ^5^, ^14^, ^15^, ^16^ Most such cases have been associated with Graves' disease indicating that both hyperthyroidism and autoimmunity can lead to pancytopenia in GD patients. Bone marrow biopsy can be either hypercellular^14^, ^15^, ^16^ or normocellular^7^ as in our patient, and potential causes for this particular association are reduced peripheral circulation time, spleen sequestration, stem cell dysfunction, and nutritional deficiencies. Since there is improvement in blood cell counts with normalization of thyroid status, thyroid hormones most probably have a direct effect on the blood cell count decline. The main challenge for our patient was his treatment, knowing that methimazole itself can cause agranulocytosis, and regarding radioiodine ablation, there was a risk of a thyrotoxic crisis. However, the incidence of pancytopenia with thionamides is rare and the incidence of agranulocytosis with the anti‐thyroid drug is 0.1%–0.5%.^18^ Therefore, we preferred to initially treat the patient with methimazole for 3 months and then commence radioiodine ablation. We periodically followed the patient's hematological counts during treatment with methimazole and radioiodine. As in all other reported cases, our patient's pancytopenia resolved after the correction of thyrotoxicosis. Case reports of Grave's disease whom also presented as pancytopenia have been summarized in Table 2.

## 4 CONCLUSION

In patients with pancytopenia and normal bone marrow, GD can be considered a missed diagnosis, as patients with GD‐induced pancytopenia respond well to anti‐thyroid drugs. Hence, alongside following hematological parameters the main aim of treatment should be to reach a euthyroid state.

## AUTHOR CONTRIBUTIONS

**Neda Meftah:** Investigation; validation; writing – original draft. **Sadegh Sedaghat:** Investigation; validation; writing – original draft. **Soghra Rabizadeh:** Supervision; validation; visualization. **Sara Seifouri:** Visualization; writing – review and editing. **Khatereh Nasirimeh:** Investigation; writing – original draft. **Manouchehr Nakhjavani:** Project administration; supervision; validation.

## FUNDING INFORMATION

This case report has no funding to declare.

## CONFLICT OF INTEREST STATEMENT

The authors do not have any conflicts of interest.

## CONSENT STATEMENT

A written informed consent (based on the patient consent policy of the Clinical Case Reports journal) was obtained from the patient.

## Data Availability

The data incorporated in this study are available from the corresponding author upon request.
